# US-Based Deep Learning Model for Differentiating Hepatocellular Carcinoma (HCC) From Other Malignancy in Cirrhotic Patients

**DOI:** 10.3389/fonc.2021.672055

**Published:** 2021-06-08

**Authors:** Hang Zhou, Tao Jiang, Qunying Li, Chao Zhang, Cong Zhang, Yajing Liu, Jing Cao, Yu Sun, Peile Jin, Jiali Luo, Minqiang Pan, Pintong Huang

**Affiliations:** Department of Ultrasound, The Second Affiliated Hospital, Zhejiang University School of Medicine, Hangzhou, China

**Keywords:** hepatocellular carcinoma, cirrhosis, deep learning—artificial neural network, ultrasonography, contrast enhanced magnetic resonance imaging

## Abstract

The aim was to build a predictive model based on ultrasonography (US)-based deep learning model (US-DLM) and clinical features (Clin) for differentiating hepatocellular carcinoma (HCC) from other malignancy (OM) in cirrhotic patients. 112 patients with 120 HCCs and 60 patients with 61 OMs were included. They were randomly divided into training and test cohorts with a 4:1 ratio for developing and evaluating US-DLM model, respectively. Significant Clin predictors of OM in the training cohort were combined with US-DLM to build a nomogram predictive model (US-DLM+Clin). The diagnostic performance of US-DLM and US-DLM+Clin were compared with that of contrast enhanced magnetic resonance imaging (MRI) liver imaging and reporting system category M (MRI LR-M). US-DLM was the best independent predictor for evaluating OMs, followed by clinical information, including high cancer antigen 199 (CA199) level and female. The US-DLM achieved an AUC of 0.74 in the test cohort, which was comparable with that of MRI LR-M (AUC=0.84, p=0.232). The US-DLM+Clin for predicting OMs also had similar AUC value (0.81) compared with that of LR-M+Clin (0.83, p>0.05). US-DLM+Clin obtained a higher specificity, but a lower sensitivity, compared to that of LR-M +Clin (Specificity: 82.6% *vs.* 73.9%, p=0.007; Sensitivity: 78.6% *vs.* 92.9%, p=0.006) for evaluating OMs in the test set. The US-DLM+Clin model is valuable in differentiating HCC from OM in the setting of cirrhosis.

## Introduction

Hepatocellular carcinoma (HCC) is the most frequent primary liver cancer type with an increasing incidence on a global-scale level (1). Cirrhosis is a well-defined high risk factor for predicting occurrence of HCC (2). Several international guidelines recommend that typical features on contrast enhanced computed tomography (CT)/magnetic resonance image (MRI) are reliable for diagnosing HCC in the setting of cirrhosis without the need for invasive biopsy procedures (3–5). However, other malignancies (OM), including intrahepatic cholangiocarcinoma (IHCC) or metastasis also develop in cirrhotic patients. Since distinct prognosis exist between these two entities, contrast imaging criteria play an important role in differentiating HCCs from OMs and guiding further treatment strategy. However, it is reported that in the setting of cirrhosis, some OMs may display similar CECT/CEMRI enhancement features as HCC, thus making them difficult to be characterized accurately (6). On the other hand, due to the unbalanced distribution of medical resources, time-consuming issues or radiation concern, the usefulness of CECT/CEMRI in evaluating focal liver lesion (FLL) is relatively limited in the clinical field.

Ultrasonography (US) is preferred as a convenient imaging modality for visualizing FLL in cirrhotic patients with cost-effectiveness and radiation-free safety (7). However, some researchers pointed out overlapping US features between HCCs and OMs (8). In addition, interpretation of US features is operator-dependent and objective, which contribute to further inter-reader variation (9). By incorporating computational methods and taking advantage of large volumes of complex digital data from imaging modalities, deep learning model (DLM) provides and uncovers much more quantitative disease characteristics that fail to be detected by naked eyes (10). In contrast to traditional radiomics, DLM automatically begin to learn information embedded in neural nets’ hidden layers from imported imaging data, and thus they do not require object segmentation and following feature extraction (11). US-DLM has been applied in various disease with promising results, including liver fibrosis (12) and thyroid cancer metastasis (13).

To our best knowledge, there was no previous study utilizing US-DLM in characterizing malignancies in cirrhotic liver. Therefore, the aim of this study was to explore the role of US-DLM in differentiating HCCs from OMs in the setting of cirrhosis.

## Materials and Methods

This retrospective study was approved by the ethics committee of local hospital with waived informed consent of each patient (Approval number: 2021-0033) and the study was conducted in accordance with the Declaration of Helsinki. But written consent was obtained before surgery or biopsy for each patient.

### Patient Enrollment

**Figure 1:**
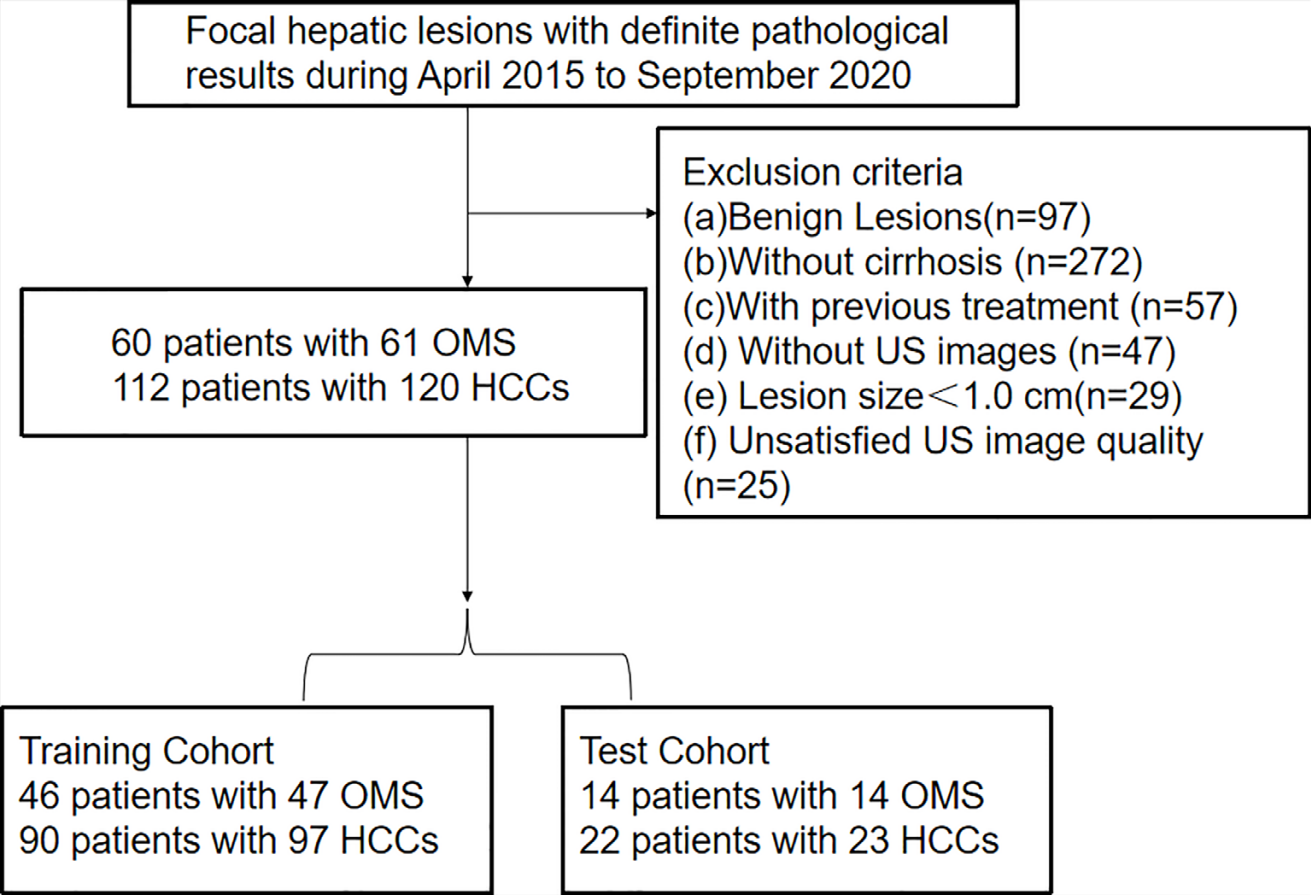
Flowchart of patient selection for differentiating hepatocellular carcinoma and other malignancy in c4irrhotic liver. Clin, clinical features; DLM, deep learning model; LR-M, liver imaging and reporting system category.

From April 2015 to September 2020, a consecutive of patients with definite pathological results of non-cystic FLL were registered. The exclusion criteria were as followings: (a) benign lesions (n=97); (b) without cirrhosis (n=272); (c) with previous treatment (n=57); (d) without US images (n=47); (e) lesion size < 1.0 cm (n=29); (f) unsatisfied US image quality (n=25); Finally, we registered 60 patients with 61 OMs and 112 patients with 120 HCCs in the present paper (**Figure 1**).

### Clinical Information

The results of serum biomarkers within 1 week before surgery or biopsy were collected. If the values of cancer antigen 199 (CA199)>37U/ml, alanine transaminase>35U/ml, aspartate transaminase>45U/ml and albumin level < 35mg/ml, then they were regarded as positive.

### US and CEMRI Examination

US and MRI examinations were carried out 7-10 days before biopsy and surgery. All US examinations were performed by three experienced board-certified radiologists, with more than 5 years’ experience in liver ultrasound imaging. All US examinations were performed with ultrasound machines, including ESAOTE (MyLab 90 X-vision, Italy), Aplio 500 (Toshiba Medical Systems, Tokyo, Japan) and Resona 7 (Mindray, Shenzhen, China) with corresponding probes. The image settings, including the time-gain compensation, the focal position, the dynamic range, and the mechanical index, were optimized for each examination according to manufacturer’s suggestion. It is routinely required to acquire images of the largest transverse cross-section and the largest long axis cross-section of the target FLLs for subsequent analysis.

Gadoxetic acid–enhanced MRI (EOB-MRI) was carried out on a 3.0-T MR scanner (Discovery MR 750; GE Healthcare, Waukesha, WI) with 0.025 mmol/kg of EOB (Primovist; Bayer AG) injection at the rate of 2 mL/s followed by 25-ml saline flush. Liver MR imaging protocol consisted of in-phase and opposed-phase T1-weighted imaging, FSE T2-weighted imaging with fat suppression, and diffusion-weighted imaging. To obtain T1-weighed arterial, portal venous, and transitional phase images, the delay time of 15 to 18 s and 50 to 60 s, 180 s were acquired, respectively, after contrast injection using volumetric interpolated breath-hold examination (VIBE) sequence. Hepatobiliary phase imaging was completed 20 min after the contrast injection.

### Interpretation of US and CEMRI Features

Two experienced radiologists reviewed all US images, who were not participated in the image acquisition and blinded to clinical information and final diagnoses of each patient. The review for US features was in concordance with a prior study (14). In brief, echogenicity was defined as mixed, hypoechoic or hyperechoic when comparing with the echogenicity of surrounding parenchyma. The shape of the lesion was deemed as round/oval or irregular. In addition, the lesion’s margin was categorized as well defined or poorly defined and halo sign was categorized as presence and absence. Regarding color Doppler images, intratumoral vascularity was categorized as absent (no vessel within the mass) or present (vessel segment within the mass).

When it comes to CEMRI images review, two other clinical-information-blinded and experienced radiologists read all cases according to the criteria defined by the version 2018 MRI-liver imaging and reporting system (LI-RADS) released by American College of Radiology (ACR). The FLL was categorized according to presence of major [arterial phase hyperenhancement (APHE), threshold growth, washout and enhancing ‘capsule’] and ancillary features (i.e. corona enhancement, restricted diffusion, etc.) proposed by CEMRI LI-RADS (15). If a FLL showed LR-M specific features (i.e. progressive central enhancement, rim APHE, targetoid restriction, etc.), then it was regarded as OM since this category was mainly indicative of other malignancies with non-HCC origin (15).

### US-DLM Model

**Figure 2:**
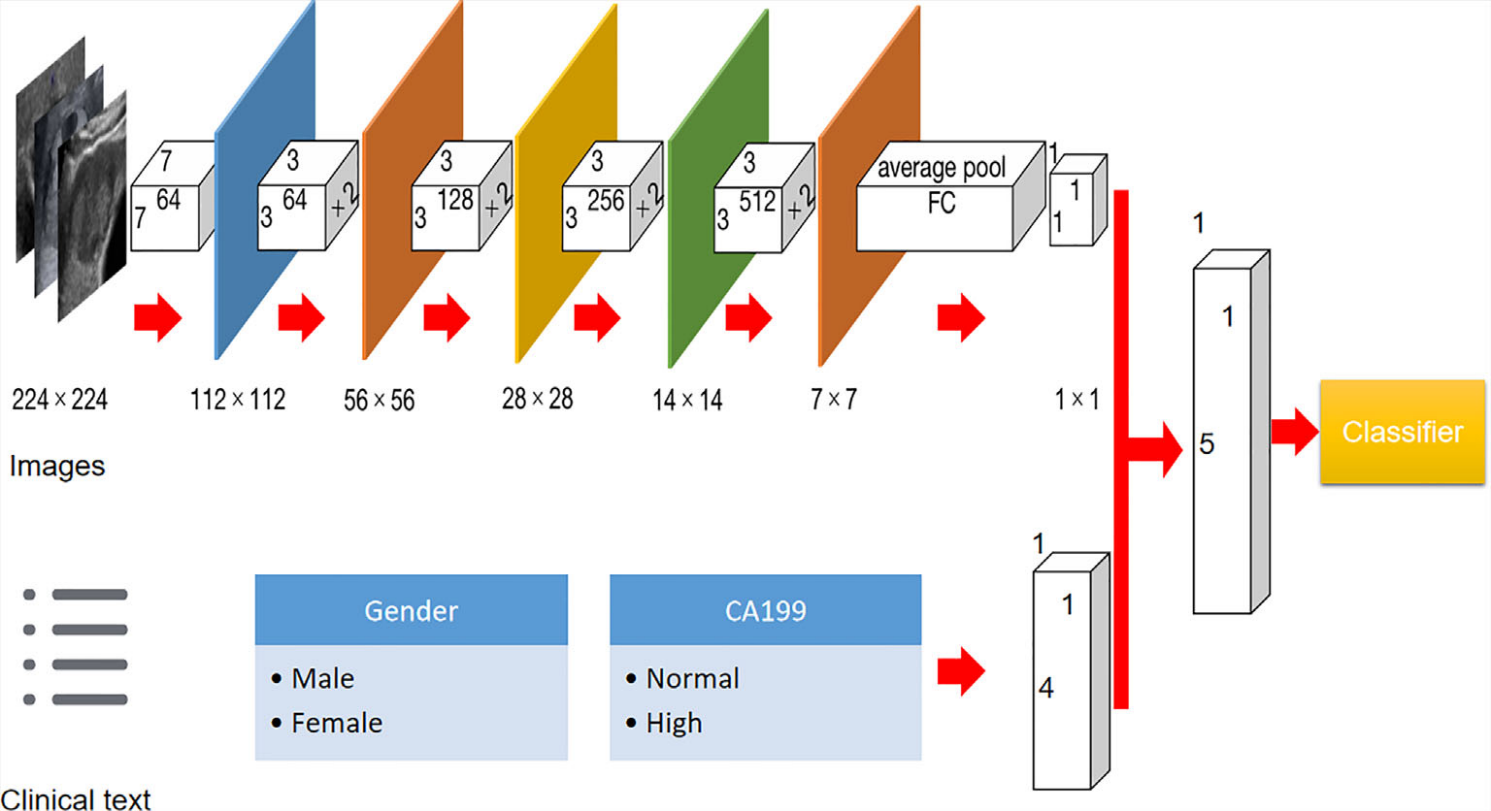
Flowchart of development of US-based Resnet model for differentiating hepatocellular carcinoma and other malignancy in cirrhotic liver.

The whole study population was split as a training and test set on a ratio of 4:1. In addition, 25% of training images were randomly chosen to form an internal validation cohort to guide the choice of hyper parameters. Before the training procedure, we applied data augmentation to avoid potential bias caused by the unbalanced data for binary classification through a number of random transformations. This method increased the diversity of training set and decreased the overfitting of the generated radiomic model. In the present paper, Resnet 18 was utilized as the base model with more than a million images pre-trained on Imagenet. It has an 18-layer convolutional neural network. In brief, the DLM model consisted of two steps: the forward computation and the backward propagation (16). The detailed flowchart and information of the DLM are illustrated in **Figure 2**. Before deep learning, the rectangular ROIs were cropped from raw US images by an experienced radiologist and resized to 224 × 224 pixels and normalized. To obtain optimal parameters, the Resnet 18 model were further fine-tuned during the training phase of our study. The learning rate was first set as 0.01, and the adjustment of learning rate can be followed by the underfitting or overfitting status of the modeling. The stochastic gradient descent (SGD) method is applied as the model optimizer. The used batch size and epoch number are both 500 in our study. The binary cross-entropy is the loss function applied in our study. The final fully connected layer was replaced by a dropout layer, a batch normalization layer, and a fully connected layer to obtain the final predictive score.

### Gold Standard

Pathological results were performed by an experienced pathologist in this institution. If a patient received both ultrasound-guided core needle biopsy (CNB) and surgical excision, then the pathological results from surgery specimen were finally regarded as the gold standard to reduce the sample bias. In our patient cohort, 85 patients with 91 HCCs and 49 patients with 50 OMs were surgery-confirmed cases, whereas the others were CNB-confirmed cases.

### Statistical Analysis

The data analyses were performed by R software (version 0.21.2, open source Python programming language, Python Software Foundation) and SPSS software (version 22.0, IBM Corporation, Armonk, NY). Continuous data were expressed as mean ± standard deviation and categorical data as numbers and percentages. Comparison of continuous data was carried out by student t test whereas comparison of categorical data was done by chi-square test, or Fisher’s test if applicable.

Significant clinical (Clin) and US features of the training set in the univariable analysis were finally put in the multiple logistic regression model. To build a predictive model based on DLM plus clinical information (US-DLM+Clin), all significant Clin features in the multiple logistic regression model and DLM outcomes were integrated into a nomogram in the training cohort. This procedure was performed by forward stepwise selection and odds ratios (ORs) with relative 95% confidence intervals (CIs) were also calculated to determine the relevance of all potential predictors for predicting OMs. The decision curve was plotted to determine the clinical usefulness of DLM+Clin model. The diagnostic performance of different models (Clin, MRI LR-M+Clin and DLM +Clin) for discriminating HCCs and OMs were performed by area under receiver operating curve (AUC) and corresponding sensitivity, specificity, positive predictive value (PPV) and negative predictive value (NPV) at the cutoff value. DeLong’s test was applied for the comparisons between the AUCs (17). McNemar test was applied for comparisons of sensitivity and specificity. For all statistical methods, a p-value < 0.05 indicated a statistically significant difference.

## Results

### Clinical Features in Predicting OMs

Sixty-one OMs included 54 IHCCs in 53 patients, 1 combined HCC-IHCC in one patient and 6 metastasis in 6 patients. When it comes to clinical information, in the training cohort, female sex and high CA199 level were more likely to be present in patients with OMs by univariate analysis (**Supplemental Table 1**). In the multivariable regression model, high CA199 level was the best valuable factor (OR=21.52) indicative of OMs, followed by female (OR=3.69) (**Supplemental Table 2**). But when including deep learning characteristics, the US-DLM became the top-ranked independent parameter for predicting OMs with an OR value of 29.52 (**Table 1**).

**Table 1:** Multivariable analysis of clinical features and US-DLM in training cohort for predicting OM in cirrhotic liver.

Parameter	β	SD	P value	OR	95% CI
Female	1.32	0.59	0.027	3.72	1.17-11.90
High CA199 level	3.07	0.72	<0.001	24.85	6.10-101.25
DLM	3.39	0.59	<0.001	29.52	9.21-95.46

HCC, hepatocellular carcinoma; OM, other malignancy; CA199, cancer antigen 199; DLM, deep learning model.

Qualitative variables are expressed as n (%) and quantitative variables are expressed as Mean ± SD.

### Diagnostic Performance of US-DLM, US and MRI LRM

On a per-lesion level, the US-DLM model yielded modest to optimal diagnostic performance with AUC of 0.84 (95%CI: 0.76-0.90), 0.67 (95%CI: 0.47-0.83) and 0.74 (95%CI: 0.57-0.87) for the internal training, internal validation and test cohort, respectively. In contrast, only absence of hyperechogenicity showed significantly difference between HCCs and OMs in the training cohort whereas other US features did not. If we applied absence of hyperechogenicity as the standard for diagnosing OMs, it achieved limited diagnostic power with an AUC value of 0.595 in the test cohort without significantly statistical level (p=0.315). Meanwhile, detailed distributions of each MRI LI-RADS category are shown in **Supplemental Table 3**. If adopting MRI LR-M as criteria for diagnosing OM, it obtained a higher, but not significantly, AUC value compared with that of US-DLM model in the test set (0.84 *vs.* 0.74; p=0.35) (**Supplemental Table 4**). Although US-DLM had a better specificity (91.3%), this model had a lower sensitivity of 57.1% (95% CI: 28.9%-82.3%) in comparison with those of MRI LR-M (sensitivity: 85.7%; specificity: 82.6%).

### Diagnostic Performance of Different Predictive Models

**Figure 3:**
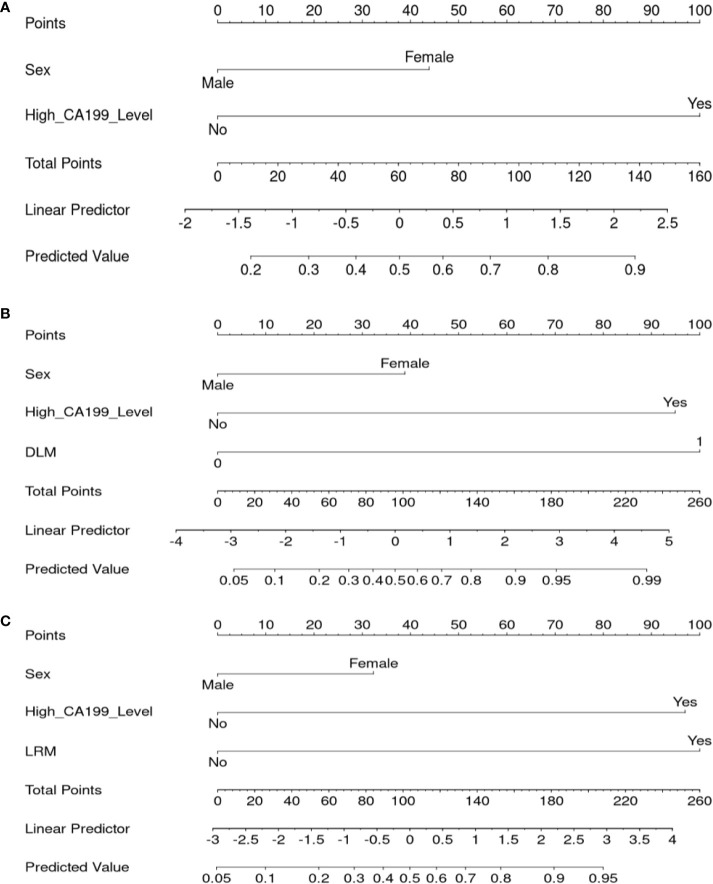
Nomogram for the Clin **(A)**, US-DLM+Clin **(B)** and MRI LR-M **(C)** model for predicting probability of OM. DLM 0: HCC, 1: OM; Clin, clinical features; DLM, deep learning model; MRI, Magnetic Resonance Imaging; LR-M, liver imaging and reporting system category; CA199, cancer antigen 199; US, ultrasonography.

**Figure 4:**
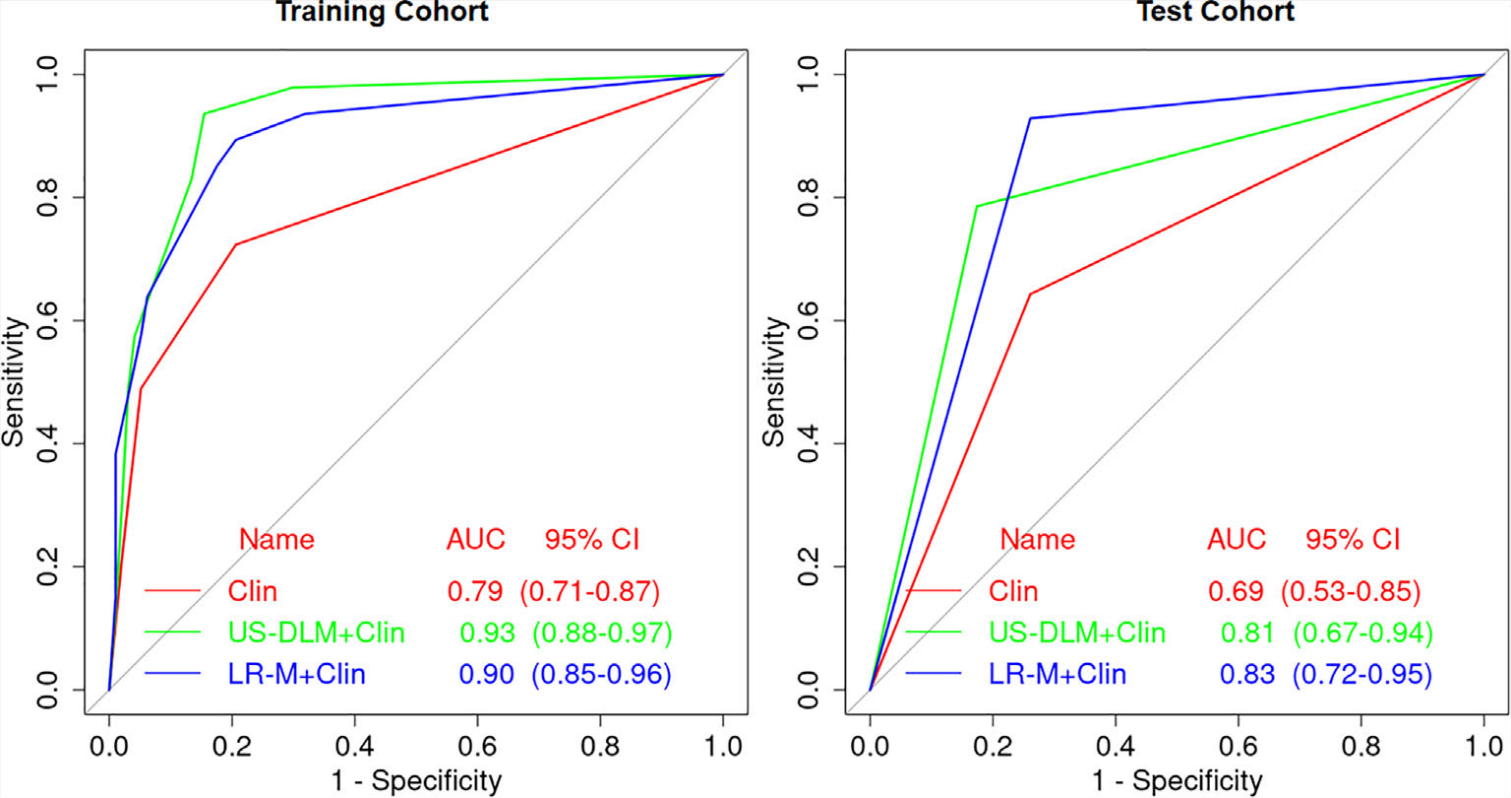
Receiver operating characteristic curves of different predictive nomogram models for predicting OM in training and test cohort in cirrhotic liver. Clin, clinical features; DLM, deep learning model; LR-M, liver imaging and reporting system category.

The nomogram graphics of Clin, US-DLM+Clin and LR-M+Clin models are shown in **Figure 3**. After adding significant clinical information mentioned above, the US-DLM+Clin nomogram model was developed, as demonstrated in **Figure 4**, with an excellent AUC of 0.93 (95%CI: 0.89-0.97) and correspondingly high sensitivity (93.6%) and specificity (84.5%) for diagnosing OMs in the training population. In the test cohort, the US-DLM+Clin also achieved optimal diagnostic performance with an AUC value of 0.81 (95%CI: 0.67-0.94). On the other hand, the Clin and LR-M+Clin had suboptimal and good diagnostic power with AUC value of 0.69 and 0.83, respectively, in the test population (**Table 2**). It should be mentioned that AUC values among three predictive models did not indicate significant differences.

**Table 2:** Diagnostic performance of different models for predicting OM in training and test cohort in cirrhotic liver.

Model		AUC	SEN (%)	SPE (%)	PPV (%)	NPV (%)
Clin	Training	0.79 (0.71, 0.87)	72.3 (57.4, 84.4)	79.4 (70.0, 86.9)	82.1 (63.1, 93.9)	79.3 (70.8, 86.3)
	Test	0.69^#^ (0.53, 0.85)	64.3 (35.1, 87.2)	73.9^*^ (51.6, 89.8)	60.0 (32.3, 83.7)	77.3 (54.6, 92.2)
US-DLM +Clin	Training	0.93 (0.888-0.97)	93.6 (82.5-98.7)	84.5 (75.8-91.1)	74.6 (61.6-85.0)	96.5 (90.0-99.3)
	Test	0.81 (0.67-0.94)	78.6 (49.2-95.3)	82.6 (61.2-95.0)	73.3 (44.9-92.2)	86.4 (65.1-97.1)
MRI LR-M +Clin	Training	0.90 (0.85-0.96)	89.4 (76.9-96.5)	79.4 (70.0 -86.9)	67.7 (54.7-79.1)	93.9 (86.3-98.0)
	Test	0.83^#^ (0.72-0.95)	92.9 (66.1-99.8)	73.9^*^ (51.6-89.8)	68.4 (43.4-87.4)	94.4 (72.7-99.9)

OM, other malignancy; AUC, area under receiver operating characteristic curve; SEN, sensitivity; SPE, specificity; PPV, positive predictive value; NPV, negative predictive value; Clin, clinical features; MRI, Magnetic Resonance Imaging; LR-M, liver imaging and reporting system category M; I-Training, internal training; I-Validation; internal validation

Numbers in parentheses are 95% confidence intervals.

*indicating significant difference compared to that of US-DLM in test cohort.

^#^indicating no significant difference compared to that of US-DLM in test cohort.

On the other hand, the US-DLM+Clin had a significantly higher specificity for evaluating OMs than that of Clin (82.6% *vs.* 73.9%; p=0.007) or LR-M+Clin model (82.6% *vs.* 73.9%; p=0.007). Meanwhile, US-DLM+Clin obtained similar sensitivity with that of Clin model (78.6% *vs.* 64.3%, p=0.210). But it had inferior sensitivity compared with that of LR-M+Clin model (78.6% *vs.* 92.9%, p=0.006).

### Diagnostic Robustness of US-DLM+Clin Model

**Figure 5:**
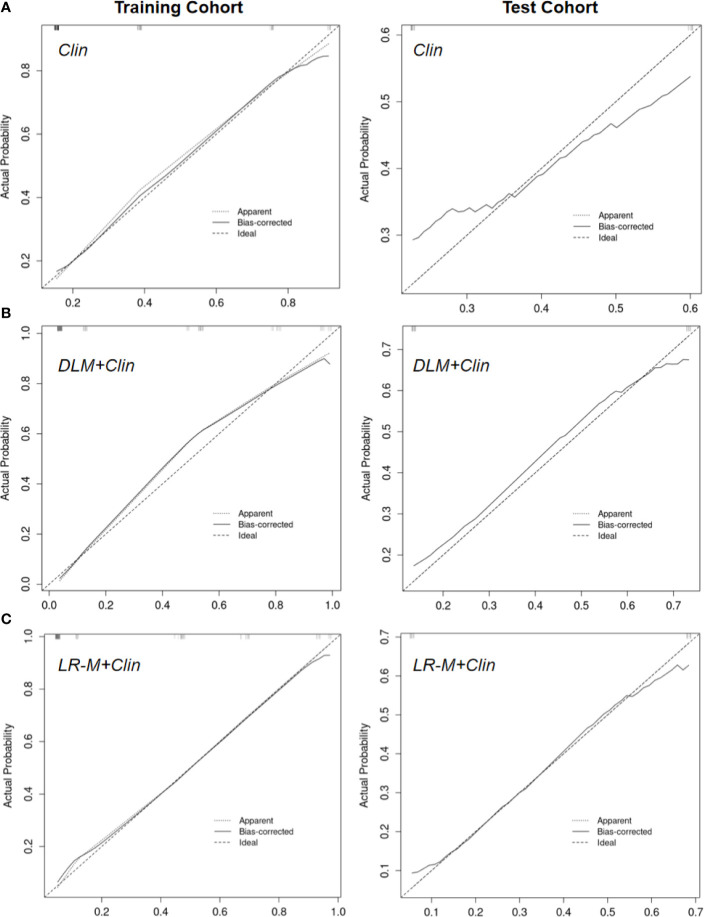
Calibration curve of Clin **(A)**, US-DLM+Clin **(B)** and MRI LR-M **(C)** model for predicting probability of OM in the training cohort and test cohort. Clin, clinical features; DLM, deep learning model; LR-M, liver imaging and reporting system category.

**Figure 6:**
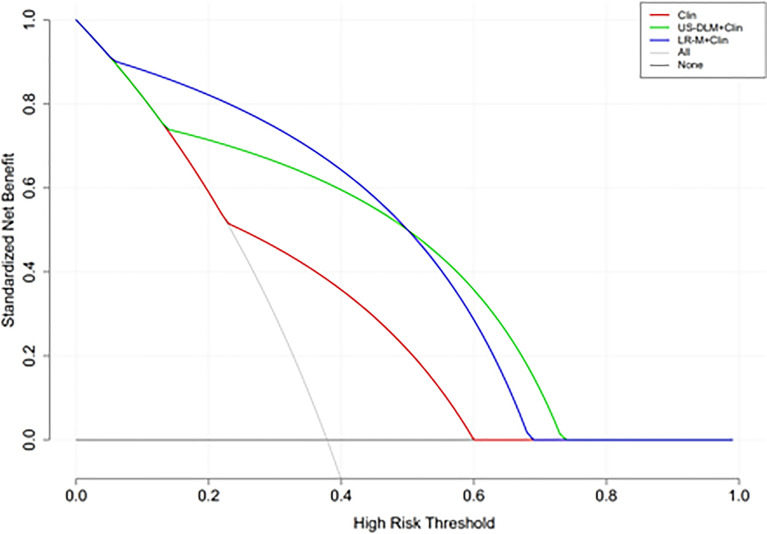
Decision curve analysis of different nomogram models for predicting probability of OM in the test cohort. The y-axis represents net benefit. The dark and grey lines measure the benefit of using the “all OMs” and “all HCCs” strategies, respectively. Clin, clinical features; DLM, deep learning model; LR-M, liver imaging and reporting system category.

The calibration curves of US-DLM+Clin correspond well between the prediction results and the observations in the training and test cohorts (**Figure 5**). The decision curves in **Figure 6** show that the US-DLM+Clin outperformed Clin model in the test cohort.

### Visualization of US-DLM

We provide representative color-pattern attention maps of HCC and IHCC, evaluated by the US, US-DLM and CEMRI (**Supplemental Figures 1** and **2**). On gray-scale US, it was difficult to confirm whether this FLL was OM or not. However, by US-DLM model, the red part inside the FLL corresponded to higher probability of OM and the blue part to lower probability. Thus, this visualization color-pattern indicated distinguishable DLM features between these two entities in cirrhotic liver.

## Discussion

There are considerable overlapping imaging features between HCCs and OMs in cirrhotic livers (18), which have distinct prognosis and treatment option (5, 19). Following suggestions from guidelines, CEMRI still remains an essential part of imaging algorithm for evaluating FLLs in cirrhosis (3). A CECT/MRI LI-RADS version was further developed in 2018 and has shown great potential in diagnosing FLLs in cirrhotic patients (18). As the criteria of the diagnosis of OMs, MRI LR-M had variable sensitivities (81.0%-89.0%) and specificities (48.0%-86.0) (18, 20), which corresponded to our results (sensitivity: 85.7%; specificity: 82.6%) in the test cohort. Hence accurate imaging-based approach in differentiation of OM and HCC in the setting of cirrhosis is an important clinical issue.

Various machine learning algorithms consist of neural networks, support vector machines (SVM) and decision tree and random forest (RF) (21). However, due to the complexity of SVM and RF, the processing require more time for training the model compared with neural networks (21). Inspired by human brain nature, convolution neural network (CNN) is the most popular DLM type in medical image analysis and can automatically identify and segment medical imaging (22). The biggest challenge in traditional machine learning and texture analysis is operator-dependently predefining region-of-interest (ROI), which could be overcome by free-hand data mining and navigating system of DLM (23). Previous literatures reported great potential of deep-learning information in differentiating FLLs based on CECT (24), CEMRI (25) and US modality (14). However, prior research groups failed to especially focus on the role of DLM in cirrhotic patients, who may have different liver cancer dynamics from normal ones (26). Meanwhile, when lesions were concurrent with cirrhosis, US had variable and suboptimal value in diagnosing HCCs with high confidence (14). Thus DLM may exist as a helpful tool to dig much more data hidden in US images than radiologists could observe. Up to our best knowledge, this paper is the first one to utilize US-based deep machine learning model to differentiate HCC and OM in cirrhotic liver.

The CNN model we used in the present study was Resnet 18, which is valuable in converting medical image information to high-throughput extraction of quantitative features (27). On US images alone, only hyperechogenicity was an independent factor for distinguishing HCC and OMs in the training cohort, which was consistent with the results of Huang et al. (8). However, when applied in the test cohort, it only showed poor diagnostic power (AUC=0.595), furthering validating the limited role of US in diagnosing FLLs. In contrast, US-DLM obtained a higher AUC value in the internal training (0.84) and test cohort (0.74), with high specificities. When compared with MRI LR-M, the specificity of US-DLM was better (91.3%) and the AUC was not statistically different (0.84 *vs.* 0.74; p=0.35) at the compensation of sensitivity loss (57.1% *vs.* 85.7%, p<0.001).

Imaging diagnostic accuracy is a clinically relevant topic because many liver lesions can display contrast-enhanced LI-RADS features resembling HCC. Clinical and epidemiological data, on the other hand, are necessary to integrate imaging features. A number of serum biomarkers and baseline clinical features have been investigated for the usefulness in diagnosing HCCs or OMs, with variable sensitivities and specificities (3). In the present paper, female and high CA199 level were independently indicative of patients with OMs, revealed by multivariate analysis. Furthermore, in the current paper we established different nomogram predictive models for OMs based on clinical information, DLM or LR-M, with the aim of improving easy-to-use and individual patient management strategy. When combing female and CA199 level together, the Clin model only manifested suboptimal diagnostic ability in the test cohort (AUC<0.70). Meanwhile, after integrating DLM features, the US-DLM +Clin model exhibited desirable diagnostic performance both in the training and test cohort (AUC=0.93 and 0.81, respectively) for predicting OMs, with DLM as the strongest determinant of OMs (OR=29.52). The US-DLM +Clin model outperformed Clin model alone according to decision curve graphics. When compared with LR-M+Clin model, US-DLM +Clin model had inferior sensitivity (78.6% *vs.* 92.9%, p=0.006) but significantly higher specificity (82.6% *vs.* 73.9%; p=0.007). Considering the fact CEMRI is more time-consuming and expensive, our results suggested that DLM based on gray-scale US images may have the great potential in differentiating OMs from HCCs in the clinical field with satisfactory cost-effectiveness.

There were several limitations in the current study. First, selection bias may exist due to single-center retrospective nature. It should be noted that specific HCC LI-RADS pattern could be detected in non-malignancy FLLs such as macronodular hepatic tuberculosis (28, 29), which deserves further attention, although we focused on hepatic malignant tumors in the current paper. Therefore a large-sample multicenter prospective study consisting of various FLLs in cirrhotic liver is urgently needed. Second, there was only one combined HCC-IHCC included in this study. Contrast enhanced imaging misdiagnosis might be present in almost two-thirds of patients with combined HCC-IHCC (30). Further study including more combined HCC-IHCCs in the OM group is required. Third, the generalizability of our findings may be limited because of exclusive enrollment of pathological-proven cases. This may explain why the OM versus HCC ratio is high in the present study (1:2), which may reflect the increasing trend of imaging-confirmed HCCs in the clinical workup. However, to eliminate any possibility of a false diagnosis based on imaging criteria alone, this selection criteria is reasonable and inevitable. In addition, false positive/negative findings may also be included in results obtained from CNB specimen. Fourth, only visible FLL on US were enrolled, therefore our US-DLM findings suffered from some degree of bias due to the missed detection of US. Fifth, a majority of enrolled cirrhotic patients were diagnosed as HBV-origin, which is common case in eastern Asia. Other causes of cirrhosis (i.e. drug toxicity or metabolic derangements) would be of interesting clinical issue when considering to apply US-DLM in the differential diagnosis. Due to limited sample size, currently, it is difficult to show the difference of US/US-DLM between the various aspects of the causative factors. Finally, rather than static images, video clips from contrast enhanced US may also serve as a promising data pool for DLM in predicting OM in cirrhosis and its utility needs validation.

In conclusion, US-DLM model is helpful in the differentiation of HCC and OM in the setting of cirrhosis. By incorporating clinical features, the combined model (US-DLM+Clin) achieves promising diagnostic performance in predicting OM, with a higher specificity than that of the MRI LR-M or MRI LR-M+Clin.

## Data Availability Statement

The original contributions presented in the study are included in the article/**Supplementary Material**. Further inquiries can be directed to the corresponding author.

## Ethics Statement

The studies involving human participants were reviewed and approved by The Second Affiliated Hospital of Zhejiang University School of Medicine. The ethics committee waived the requirement of written informed consent for participation.

## Author Contributions

PH contributed to conception and design of the study. ChZ, CoZ, PJ, JL, and MP acquired of data. HZ and TJ analyzed and interpreted data. JL and YS performed the statistical analysis. YS and JL supervised the work. HZ wrote the first draft of the manuscript. HZ and TJ revised article critically for important intellectual content. All authors contributed to the article and approved the submitted version.

## Funding

The study was supported by the National Key R&D Program of China (2018YFC0115900), National Natural Science Foundation of China (Grant No.82030048, 81527803, 81901871, 82001818), Zhejiang Science and Technology Project (LQ21H180007), and Zhejiang Science and Technology Project (No.LQ20H180009).

## Conflict of Interest

The authors declare that the research was conducted in the absence of any commercial or financial relationships that could be construed as a potential conflict of interest.
