# Supplementary material for: US-Based Deep Learning Model for Differentiating Hepatocellular Carcinoma (HCC) From Other Malignancy in Cirrhotic Patients
Source: Front Oncol. 2021 Jun 8;11:672055. doi: 10.3389/fonc.2021.672055 (PMC8217663; doi:10.3389/fonc.2021.672055)
Supplement: Supplementary file 1 [file DataSheet_1.doc]

**Supplemental information**

**Supplemental Table1. Clinical information and ultrasonographic features of HCC and OM in training and test cohort in cirrhotic liver**

| **Parameter** | **Training Cohort** | | | **Test Cohort** | | |
| --- | --- | --- | --- | --- | --- | --- |
|  | HCC(n=97) | OM(n=47) | P value | HCC(n=23) | OM(n=14) | P value |
| **Clinical information** |  |  |  |  |  |  |
| Age (y) | 58.9±11.2 | 60.2±11.1 | 0.523 | 59.8±10.4 | 63.4±8.5 | 0.278 |
| Female | 16 (17.8%) | 20(43.5%) | **﹤0.001^#^** | 4 (18.2%) | 3(21.4%) | 1.000 |
| Albumin level﹤35mg/ml | 17(18.9%) | 15(32.6%) | 0.089 | 6(27.3%) | 2(14.3%) | 0.441 |
| HBV as  cirrhosis origin (Yes) | 80(88.9%) | 35(76.1%) | 0.161 | 21(91.3%) | 12(85.7%) | 0.625 |
| ALT level﹥35U/L | 55(60.4%) | 20(43.5%) | 0.070 | 12(54.5%) | 7(50.0%) | 1.000 |
| AST level﹥45 U/L | 42(46.2%) | 14(30.4%) | 0.098 | 5(22.7%) | 5(35.7%) | 0.462 |
| CA199 level﹥37U/ml | 5(5.5%) | 23(50.0%) | **﹤0.001^#^** | 2(9.1%) | 8(57.1%) | **﹤0.001^#^** |
| **US features** |  |  |  |  |  |  |
| Nodule Size(mm) | 46.4±28.8 | 48.1±28.7 | 0.523 | 44.4±14.8 | 46.2±26.0 | 0.783 |
| Hyperechogenicity | 29 (29.9%) | 5(10.6%) | **0.012^#^** | 6 (26.1%) | 1(7.1%) | 0.217 |
| Ill-defined margin | 65 (67.0%) | 32(68.1%) | 1.000 | 16 (69.6%) | 9(64.3%) | 1.000 |
| Irregular shape | 51 (52.6%) | 27(57.4%) | 0.280 | 11 (47.8%) | 9(64.3%) | 0.498 |
| Halo sign (Yes) | 26 (26.8%) | 13(27.7%) | 1.000 | 6 (26.1%) | 3(21.4%) | 1.000 |
| Intratumoral vascularity(Yes) | 48 (49.5%) | 16(34.0%) | 0.107 | 10 (43.5%) | 7(50.0%) | 0.745 |

Note: HCC, hepatocellular carcinoma; OM, other malignancy; ALT, alanine transaminase; AST, aspartate transaminase; AFP, alpha fetoprotein; CA199, cancer antigen 199; US,ultrasonography

Qualitative variables are expressed as n (%) and quantitative variables are expressed as Mean±SD

**Supplemental Table 2. Multivariable analysis of clinical features in training cohort for predicting OM in cirrhotic liver**

| **Parameter** | **β** | **SD** | **P value** | **OR** | **95% CI** |
| --- | --- | --- | --- | --- | --- |
| Female | 1.31 | 0.52 | 0.011 | 3.69 | 1.34-10.13 |
| High CA199 level | 3.07 | 0.66 | ﹤0.001 | 21.52 | 5.86-79.03 |

Note: HCC, hepatocellular carcinoma; OM, other malignancy; cancer antigen 199; OR, odds ratio; CI, confidence level

**Supplemental Table 3. Distribution of MRI LI-RADS category in training and test cohort**

| Parameter | Training Cohort | | Test Cohort | |
| --- | --- | --- | --- | --- |
|  | HCC(n=97) | OM(n=47) | HCC(n=23) | OM(n=14) |
| MRI LR 3/4 | 6 (6.2%) | 4 (8.5%) | 0 (0) | 1 (7.1%) |
| MRI LR 5 | 75 (77.3%) | 6 (12.8%) | 19 (82.6%) | 1 (7.1%) |
| MRI LR-M | 16 (16.5%) | 37 (78.7%) | 4 (17.4%) | 12 (85.7%) |

Note: HCC, hepatocellular carcinoma; OM, other malignancy; MRI, Magnetic Resonance Imaging; LR-M, liver imaging and reporting system category M

Qualitative variables are expressed as n (%)

**Supplemental Table 4 Diagnostic performance of DLM and MRI LI-RADS category in training and test cohort**

| **Model** |  | **AUC** | **SEN (%)** | **SPE (%)** | **PPV (%)** | **NPV (%)** |
| --- | --- | --- | --- | --- | --- | --- |
| US-DLM | I-Training | 0.84  (0.76-0.90) | 77.5  (61.5-89.2) | 90.7  (81.7-96.2) | 81.6  (65.7-92.3) | 88.3  (79.0-94.5) |
|  | I-Validation | 0.67  (0.47-0.83) | 57.1  (28.9-82.3) | 77.3  (54.6-92.2) | 44.4  (13.7-78.8) | 85.0  (62.1-96.8) |
|  | Test | 0.74  (0.57-0.87) | 57.1  (28.9-82.3) | 91.3  (72.0-98.9) | 80.0  (44.4-97.5) | 77.8  (57.7-91.4) |
| MRI LR-M | Training | 0.81  (0.74-0.87) | 78.7  (64.3-89.3) | 83.5  (74.6-90.3) | 69.8  (55.7-81.7) | 89.0  (80.7-94.6) |
|  | Test | 0.84^#^  (0.68-0.94) | 85.7*  (57.2-98.2) | 82.6*  (61.2-95.0) | 75.0  (47.6-92.7) | 90.5  (69.6-98.8) |

**Note:** AUC, area under receiver operating characteristic curve; SEN, sensitivity; SPE, specificity; PPV, positive predictive value; NPV, negative predictive value; MRI, Magnetic Resonance Imaging; LR-M, liver imaging and reporting system category M; I-Training, internal training; I-Validation; internal validation

Numbers in parentheses are 95% confidence intervals

* indicating significant difference compared to that of US-DLM in test cohort

# indicating no significant difference compared to that of US-DLM in test cohort

**Supplemental figure 1**. A 64-years-old male cirrhotic patient, HBV(+). On conventional US (a), the 86.2mm lesion shows mixed echogenicity; On US-DLM model (b), the lesion mainly consists of blue parts, indicating a higher possibility for HCC; On T2-weightd image (c), the lesion demonstrates mixed-intensity signal; On CEMRI, the lesion shows partial arterial phase enhancement (d) and washout and enhancing ‘capsule’, categorized as CEMRI LR-5 (e); A HCC diagnosis is confirmed by the histological specimen after surgery (f).

**Supplemental figure 2**. A 67-years-old alcoholic cirrhotic patient, male, HBV(-). On conventional US (a), the 45.0mm lesion shows hypo-echogenicity; On US-DLM model (b), the lesion mainly consists of red parts, indicating a higher possibility for OM; On T2-weightd image (c), the lesion demonstrates hyper-intensity signal; On CEMRI, the lesion shows no arterial phase enhancement (d) and progressive central enhancement, categorized as CEMRI LR-M (e); An IHCC diagnosis is confirmed by the histological specimen after surgery (f).
