# Supplementary figures and images for: US-Based Deep Learning Model for Differentiating Hepatocellular Carcinoma (HCC) From Other Malignancy in Cirrhotic Patients
Source: Front Oncol. 2021 Jun 8;11:672055. doi: 10.3389/fonc.2021.672055 (PMC8217663; doi:10.3389/fonc.2021.672055)

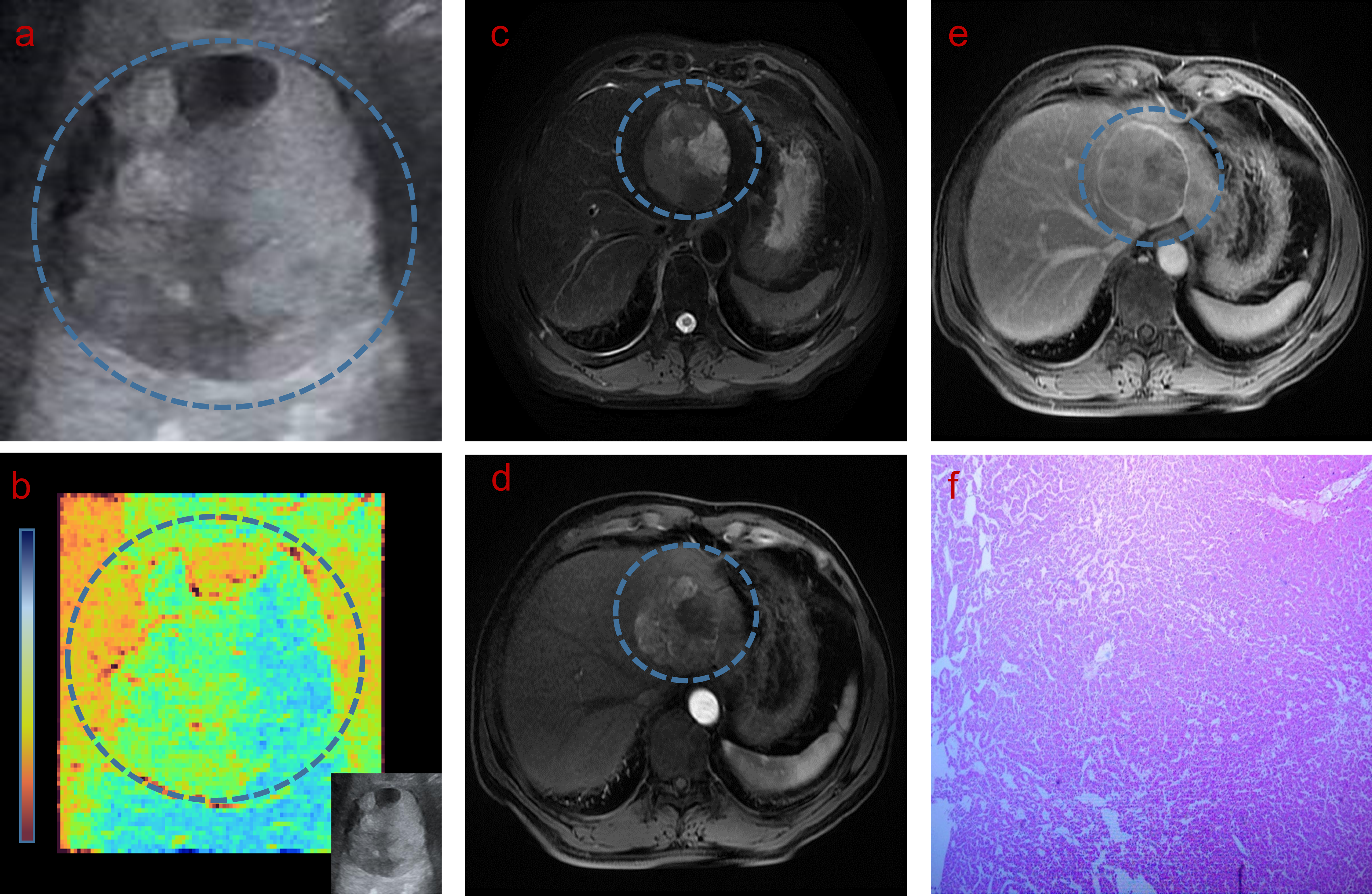

Supplement: Supplementary file 2 [file Image_1.tif]

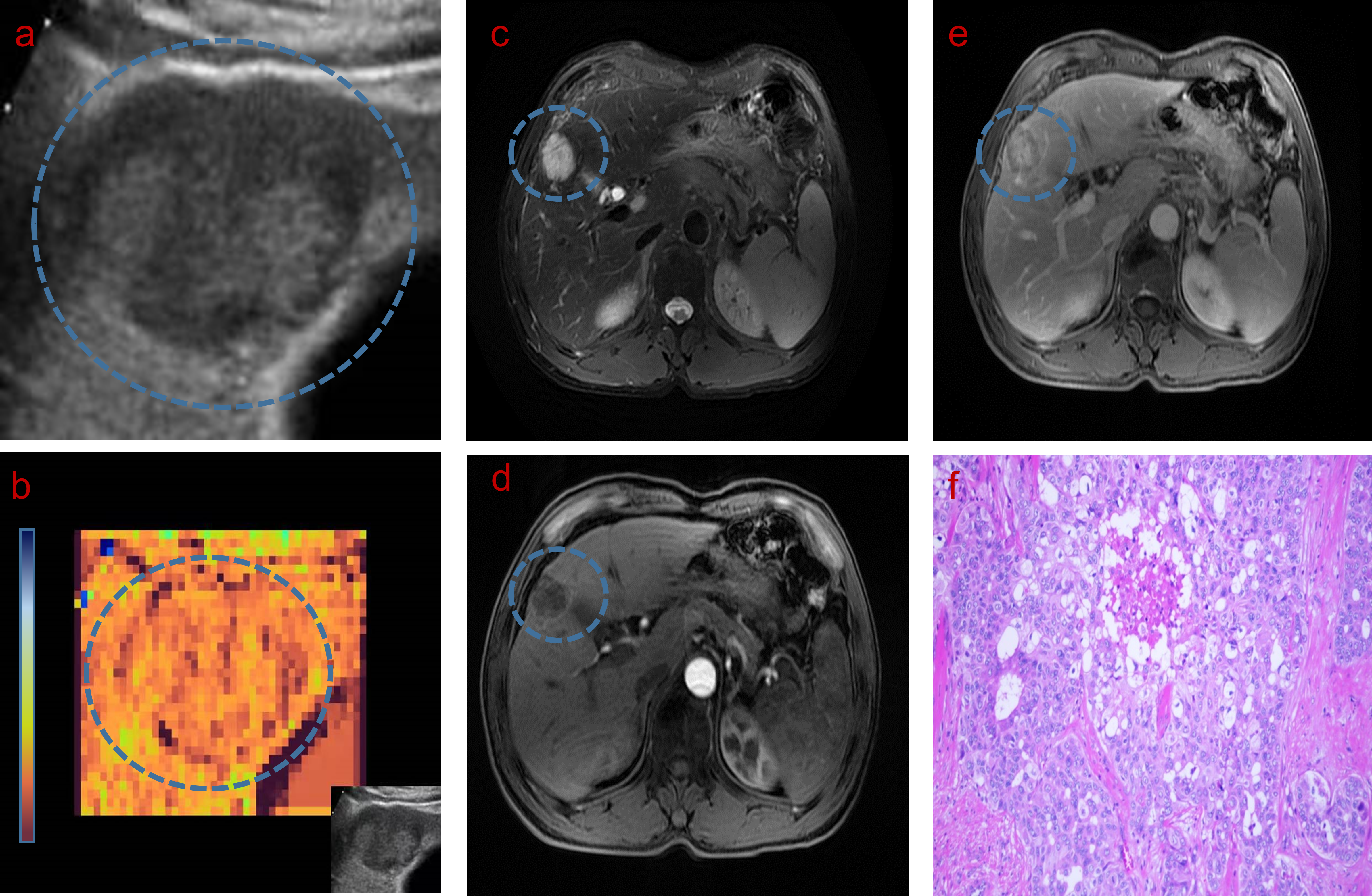

Supplement: Supplementary file 3 [file Image_2.tif]
